# Progress in Paratuberculosis Control Programmes for Dairy Herds

**DOI:** 10.3390/ani14071127

**Published:** 2024-04-07

**Authors:** Maarten F. Weber, David Kelton, Susanne W. F. Eisenberg, Karsten Donat

**Affiliations:** 1 Royal GD, P.O. Box 9, 7400 AA Deventer, The Netherlands; m.weber@gdanimalhealth.com; 2 Department of Population Medicine, University of Guelph, Guelph, ON N1G 2W1, Canada; dkelton@ovc.uoguelph.ca; 3 Animal Disease Fund of Lower Saxony, Brühlstraße 9, 30169 Hanover, Germany; 4 Thuringian Animal Diseases Fund, Victor-Goerttler-Straße 4, 07745 Jena, Germany; kdonat@thtsk.de; 5 Clinic for Reproduction and Neonatology of Animals, Justus-Liebig-University Gießen, Frankfurter Straße 106, 35392 Gießen, Germany

**Keywords:** paratuberculosis, Johne’s disease, *Mycobacterium avium* subsp. *paratuberculosis*, voluntary control, education, surveillance, financial aid, future perspectives

## Abstract

**Simple Summary:**

Paratuberculosis control programmes in countries with a relevant dairy industry differ largely in participation and progress. Despite over a century of experience with paratuberculosis control efforts, major knowledge gaps still exist, including the efficacy of control programmes and drivers and barriers influencing the uptake of control programmes amongst farmers. Biennially, the International Dairy Federation (IDF) brings together experts on paratuberculosis control to share the most recent knowledge and experiences regarding practical aspects of paratuberculosis control. Taken together, studies on control programmes presented at the 7th and 8th IDF ParaTB Fora and the 15th International Colloquium on Paratuberculosis (ICP) indicated a key finding that a reduction of the prevalence of Map infection had been achieved by various programmes. Important prerequisites for successful control were long-term stable funding, stakeholder commitment and incentives for farmers to participate. Focal topics to improve the control of Map were identified, including improved communication about the epidemiology of infection and its control, increased attention to intrauterine, calf-to-calf and adult-to-adult transmission, sound but easy-to-use surveillance schemes, measures to reduce between-herd transmission and breeding for resistance to Map infection. Research in parallel with these programmes was found to keep interest in Map control high among stakeholders and farmers and to enable programme improvement.

**Abstract:**

While paratuberculosis control has been studied for over a century, knowledge gaps still exist regarding the uptake and efficacy of control programmes. This narrative review aims to summarise studies on control programmes presented at the IDF ParaTB Fora in 2021 and 2022 and the International Colloquium on Paratuberculosis in 2022. Studies were grouped by topic as follows: successful control, field studies, education and extension, voluntary and compulsory control programmes, and surveillance. Various Map control programmes resulted in a decreasing animal and herd level Map prevalence. Long-term stakeholder commitment, stable funding, involvement of herd veterinarians and incentives for farmers to participate were shown to be pivotal for long-term success. Control measures focused on vertical and calf-to-calf transmission may improve Map control in infected herds. Easy-to-capture visualisation of surveillance test results to inform participants on the progress of Map control in their herds was developed. The probability of freedom from disease and estimated within-herd prevalence were identified as good candidates for categorisation of herds to support low-risk trade of cattle. Results of the surveillance schemes may inform genetic selection for resistance to Map infection. In conclusion, successful paratuberculosis control is feasible at both the herd and country level provided that crucial prerequisites are met.

## 1. Introduction

Paratuberculosis (Johne’s disease) is a mycobacterial disease of ruminants caused by *Mycobacterium avium* subsp. *paratuberculosis* (Map). It starts as a localised infection that may become systemic and often results in chronic granulomatous enteritis, leading to weight loss, diarrhoea (in some species) and eventually death [1].

Paratuberculosis is of concern to dairy cattle industries worldwide because of its economic impact on dairy production [1,2,3] and uncertainty about the potential involvement of Map in human disease [4,5]. Concerted actions to control Map infection in ruminants have been taken from the 1920s onwards [6]. Stated primary aims of existing control programmes include improving animal health and welfare, reducing production losses, maintaining trade on both regional and international levels as well as reducing potential hazards to public health [1]. Recent reviews indicate that, since then, control programmes for paratuberculosis have been developed in at least 22 countries [1,7]. Control efforts include vaccination, test-and-cull as well as taking preventive management measures to reduce the risk of Map introduction into herds and flocks, as well as the spread of the infection within infected herds and flocks.

Despite more than 100 years of experience with concerted control efforts against paratuberculosis, major knowledge gaps still exist [8]. These knowledge gaps include the development of Map prevalence in time and space, genetic and environmental factors influencing the susceptibility of ruminants to infection and transmission of Map, the role of wildlife in Map transmission, efficacy of vaccines, efficacy of control programmes as well as factors influencing the long-term commitment of farmers to control Map and participate in control programmes [8].

Given these knowledge gaps, it is pivotal to globally share information and experiences from existing control programmes in various countries. To facilitate this knowledge transfer, the International Dairy Federation (IDF) organises ‘Paratuberculosis Fora’ as a satellite meeting to the ‘International Colloquium on Paratuberculosis’ organised by the International Association for Paratuberculosis.

To facilitate the dissemination of this knowledge to a wider audience of stakeholders, decision makers and researchers involved in paratuberculosis control, the aims of this narrative review are to summarise developments on Map control programmes presented at recent IDF Paratuberculosis Fora or at a recent International Colloquium on Paratuberculosis and to discuss future perspectives of Map control. Studies eligible for inclusion in this narrative review were presented either in the ‘control programs and education’ session at the 15th International Colloquium on Paratuberculosis (Dublin, 13 June 2022) or at the 7th (online, 26 May 2021) or 8th IDF Paratuberculosis Forum (Dublin, 12 June 2022) or were published in a Special Issue of the ‘IDF Bulletin 523/2023 Proceedings of the 7th and 8th IDF Paratuberculosis Forum (March 2023)’. In addition, a search on Pubmed (https://pubmed.ncbi.nlm.nih.gov/ accessed on 20 February 2023) was performed to identify full papers that resulted from these studies presented, using author names and ‘paratuberculosis’ as keywords. If identified, such papers were eligible for inclusion in this review as well. Studies were grouped regarding topic. Seven topics were distinguished: successful control, field studies, education and extension, voluntary control programmes, financial aid and compensation, compulsory control programmes, and surveillance in test-negative (meta) populations. In this review, a control programme was considered ‘compulsory’ if there was a legal requirement for farmers to participate or to act. All other control programmes were considered ‘voluntary’, even if farmers were required to participate by private bodies or companies such as milk processors.

## 2. Successful Control: The Example Set by Norway

An example of successful control in a country was set by Norway. This country, not an EU member, has implemented mandatory control measures at the national level. Government legislation enforces the control of paratuberculosis and includes restrictions on animal movements [9] with the aims of eradicating Map from Norway and subsequently avoiding the re-introduction of Map [1]. A national surveillance programme for paratuberculosis was established in Norway in cattle in 1996 and was extended to llamas and alpacas in 2000, goats in 2001, and sheep in 2002 [10,11]. An important part of the control activities and a good example of successful paratuberculosis control was the eradication programme named “Healthier goats”, run by the Norwegian Goat Health Services and the dairy organisation (TINE) between 2001 and 2015. A large proportion of Norwegian goat herds underwent this programme and reduced the prevalence of Map infection to below detection limits [12]. 

Since 2016, paratuberculosis has not been observed in Norway. More than 12,300 faecal and organ samples were tested for Map between 2016 and 2021 without any positive result, and no Map-positive animals from Norway have been reported since 2017. Furthermore, 3996 bulk-milk samples from cattle herds were tested for Map antibodies without any positive result [12]. The country tightened biosecurity measures and monitoring of the currently Map-free metapopulation. Vaccination, which was previously used to control paratuberculosis, is now prohibited. Faecal samples are collected at farms for active surveillance. Furthermore, organ samples are collected for passive clinical surveillance of suspicious cases on farms or at slaughterhouses [12]. 

In the near future, Norwegian activities will focus on prevention of new introductions of Map. For this, the geographical longitude of the country in northern Europe with a land border exclusively with Sweden, Finland and Russia is advantageous. In Norway, the introduction of cattle from other countries is very limited, while semen and embryos are imported for breeding. Imports of animals of susceptible species, including llamas and alpacas, as well as the import of animal feed (e.g., hay or silage), are still regarded as risks for the re-introduction of Map [12]. Like Norway, Sweden has had mandatory regulations in force since 1998. Suspicion of clinical disease must be reported in all species; active monitoring includes all adult cattle submitted for necropsy. Elimination of an infected herd (stamping out) together with extensive tracing of all contact herds is mandatory [1].

The mandatory eradication programmes in Norway and Sweden have made it possible to reduce the occurrence of Map in the herds or flocks located in these countries to a level where Map is not detectable anymore [1,12,13]. Apparently, Norway and Sweden are the only countries in the world that can be considered paratuberculosis-free based on their functional active and passive surveillance system for paratuberculosis without detection of any positive cases for several years.

## 3. Field Studies

Field studies presented were focused on the epidemiology, diagnostics and economics of Map control.

In Canada, control efforts are, in general, organised at the provincial level. Canada-wide, testing a set of six environmental faecal samples per farm for Map prevalence resulted in relevant differences between regions, with true prevalence estimates between 24 and 66% [14]. Testing a single composite ‘super’ swab gave a positive result in 10 out of 15 herds with ≥1 positive environmental sample [15]. 

In Ontario, Canada, bulk milk testing was used to classify herds according to the relative optical density of the applied ELISA test, with 52% of herds classified as ‘low risk’, 40% as ‘moderate risk’ and 6% as ‘high risk’ herds [15]. In addition, a longitudinal study showed that the management of herds participating in 2013 and 2019 in the paratuberculosis control programme in Ontario, Canada, changed over time [16]. This included an increase in management factors related to a decreased risk (fewer herds purchasing cattle, buying cattle—if any—more frequently from a single herd and buying younger animals, keeping sick and lame cows out of the maternity pen and feeding low-risk colostrum) as well as management factors related to an increased risk (group calving, leaving calves with dams longer, feeding bulk tank milk and non-saleable milk, group housing of calves, decreased cleanliness of weaned/bred heifers and decreased cleanliness of dry and milking cow environments). These changes highlight the importance of repeated risk assessments and must be considered in the recommendations given to farmers [16]. 

In Alberta, Canada, a field study started in 2022 with a focus on herd-level Map eradication through the implementation and evaluation of farm-specific disease control strategies. This approach was proposed by the Canadian Johne’s Coordinating Committee and should serve as the starting point for a future national programme [15].

In a German study, the herd level prevalence of Map-infected dairy herds was estimated in three different regions by direct Map PCR on environmental samples. Sampling socks and liquid manure resulted in an estimated true prevalence of 14.8% of the dairy herds in ‘North’, 49.5% in ‘East’ and 3.6% in ‘South’ Germany [17]. Another German study quantified the herd-level sensitivity of testing such environmental samples and of testing pooled faecal samples. In a simulated herd with a within-herd prevalence of faecal shedders of 2%, the herd level sensitivity of a qPCR assay of 1:5 pooled faecal samples of 80 out of 300 adult cattle was determined to be 78% [18]. The herd-level sensitivity of testing pooled milk samples and environmental samples was considerably lower [19,20]. However, by including results of seven repeated monthly samplings of pooled faeces from the barn environment and testing these samples with culture as well as direct qPCR, a cumulative herd-level sensitivity of 95% could be reached in herds with a within-herd prevalence of shedders of ≥2% [19,20]. 

The true animal-level prevalence of Map infection in Austria was estimated in sheep and goats at 1.2% and 3.5%, respectively, whereas the apparent herd-level seroprevalence was estimated at 9% and 11%, respectively [21]. Observed risk factors in goat herds were herd size, dairy herd, introduction of goats from other herds, animal health service membership and the presence of farmed game [21,22]. Risk factors in sheep flocks were the introduction of sheep from other flocks and the presence of cattle or goats [21,22]. 

In the Basque country (Northern Spain), a long-term vaccination and test approach trial in 21 dairy herds was compared with a test-and-cull approach in nine herds [23,24]. In vaccinating herds, the median survival of cattle was longer, and the apparent prevalence of Map shedding decreased more rapidly compared to test-and-cull herds. However, beyond year 10 of the experiment, shedding was negligible in both groups of herds [23,24]. Therefore, the authors concluded that vaccination reduced faecal shedding and the clinical impact of the disease and enhanced the longevity of individual cattle. Due to reduced shedding and within-herd prevalence of shedders, vaccination may be considered an effective tool to control the disease in a shorter time frame [24].

## 4. Education and Extension

Knowledge about the infection-and-disease process is pivotal for successful paratuberculosis control, and therefore, training farmers and their veterinarians was identified as a crucial element of control programmes. Regarding veterinarians, the concept of ‘approved veterinary practitioners’ was reported to be a core element of the control programmes in the UK and Ireland [25,26]. The Irish approach includes a day-long extension course finishing with a knowledge test and a course attendance certificate. Only after completion are veterinary practitioners allowed to act within the national programme and to access funding provided by the programme fund, e.g., for veterinary risk assessments. Ongoing professional development for farmers is included in most of the programmes, e.g., in Italy, Germany, Canada and Australia [12,20,27,28]. In Italy, videos and brochures were used to increase disease awareness and knowledge among farmers regarding the risk of introduction and measures to control the disease at the herd level [27]. An excellent tool to bring knowledge to farmers is performing annual risk assessments for each dairy herd, as has been established in Ireland and the UK [25,26]. The discussion about the most relevant management measures following risk assessment has been shown to be very valuable in defining farmers’ goals and giving tailored recommendations to farmers. In the same way, presenting test results to farmers and benchmarking their progress in Map control over time enhances knowledge transfer to the farmers [25,26]. Excellent ideas result from these on-farm discussions, e.g., the creation of a ‘green calving line’ and a ‘green calf line’ with exclusion of test-positive cattle and their offspring from these green lines [29]. Instead of presenting academic knowledge like the established pathways of infection (in utero, colostrum/milk, faecal, adult to calf), better results were achieved by discussing the risk of spread within the herd by differentiating ‘one to one’ versus ‘one to many’ transmission causing an epidemic [29]. Furthermore, breeding replacements from low-risk cattle only and a delay in breeding high-risk cattle in seasonal calving herds may appeal to the farmers [29].

## 5. Voluntary Control Programmes

Concerted voluntary control efforts have been established in many countries since the 1940s [1,6,7]. Over time, their objectives have gradually shifted from elimination of Map towards control of Map in infected herds and flocks [1,6]. Frequently cited objectives include a reduction in the incidence of clinical paratuberculosis, a reduction in the prevalence of infection, to provide assurance to low-risk herds when buying replacement stock, as well as a reduction in Map contamination in the human food chain to safeguard market access [1].

In two neighbouring European countries, the Netherlands and Belgium, similarly designed voluntary control programmes were initiated in 2006 with the aim of reducing Map prevalence in dairy herds as well as reducing the concentration of Map in raw milk delivered to milk processing industries [30,31,32]. In line with the latter goal, the Dutch programme is designated as a Milk Quality Assurance Programme (MQAP). Participation is required by dairy processors in both countries, resulting in the participation of 99% of the Dutch [33] and 75% of Belgium dairy herds [32]. In both programmes, participating herds are assigned a herd status based on the results of herd examinations. Assigned statuses include A for test-negative herds, B for test-positive herds from which test-positive cattle are culled and C for test-positive herds in which test-positive cattle are retained, even though exact definitions and deadlines for culling test-positive cattle differ between the two countries [30,34,35]. Each herd examination consists of testing either individual milk samples of all lactating cattle or serum samples of all cattle ≥ 3 years of age by ELISA. Farmers are entitled to confirmatory faecal PCR testing of ELISA-positive cattle. Unless confirmatory testing yields a negative result, ELISA-positive animals have to be removed within a limited period of time. The interval between herd examinations depends on results of the previous herd examination: test-positive herds are tested annually, and test-negative herds biennially. Participating herds are advised to take preventive management measures to reduce the risk of introduction and within-herd spread of Map; however, a formal risk assessment and implementation of management measures are not required. Analyses of results achieved in the Dutch MQAP during a 15-year period showed that the proportion of participating herds with the preferred herd status (status A) increased to 80%. Moreover, a decreasing apparent prevalence and an increasing age at onset of test-positivity were observed. These observations indicate a reduced transmission of Map after long-lasting participation in the programme and, thus, indicate that the programme contributes to Map control [30].

To improve the health status of dairy herds and to protect the dairy export market, the Italian government issued “National guidelines for the control of bovine paratuberculosis and for assigning the health ranking of herds” in 2013 [1,27,36]. The main components of the national guidelines include passive surveillance with mandatory reporting of clinical cases and a classification of bovine herds [27]. Each herd is assigned a Map risk level (PTC to PT5) based on the presence or absence of clinical disease and on results of annual serological herd tests [27,36]. The costs of testing and culling test-positive cattle have to be paid by participating farmers. The guidelines were implemented in between 2014 and 2016 [1]. Application of the guidelines, however, varies between Italian regions [27]. The herd-level true prevalence of Map in the Italian region of Lombardy has been estimated at 70% [37]. Results observed in a seven-year period (2014–2021) following the introduction of the Map control guidelines in Lombardy showed an increasing proportion of participating dairy herds (from 56% to 81%), an increasing number of participating dairy herds being tested annually (from 7% to 31%) and decreasing proportions of test-positive herds (from 74% to 52% of the tested herds) as well as test-positive cattle (from 4% to 1%) [27]. As a result, the proportions of participating herds with the preferred lower risk levels (PT3 to 5) increased over time [27].

The UK National Johne’s Management Plan (NJMP) requires participating farmers to opt for one of six control strategies to manage Map infection on their farms. In short, the six strategies are as follows: biosecurity; improved farm management; improved farm management and strategic testing; improved farm management test and cull; breed to terminal sire; and firebreak vaccination [25,38]. The most popular strategies are ‘improved farm management and strategic testing’ (43% of participating herds in 2020), ‘improved farm management, test and cull’ (28% of herds) and ‘breed to terminal sire’ (12% of herds). The most important result was the creation of a social norm to test and manage Map infections [39]. Approximately 70% of participating herds are using regular individual cow milk ELISA testing within their control programmes, either as targeted sampling of 30 cows or quarterly testing of individual milk samples based on the Danish approach to identify and manage infected cows within their herds [38]. The UK national farm assurance programme (Red Tractor) incorporated the NJMP into the dairy farm standards in 2020, effectively making it mandatory to be part of the NJMP, resulting in 95% of all UK dairy farmers now being included in the scheme [38].

In Germany, no nationwide control programme is in place. Several federal states implemented regional Map control programmes. Voluntary regional control programmes aiming at elimination at the herd level in cattle are performed in Thuringia, Saxony and Mecklenburg-Western Pomerania. The main objectives of these programmes are a reduction in within-herd prevalence when the infection is present in a herd and prevention of the spread of paratuberculosis to uninfected herds by certification, surveillance and biosecurity measures in herds that are not affected. An initially Map-positive herd can be certified as non-suspect by repeated individual faecal culture of each cow for three consecutive years (certification phase) with only negative results [40]. Due to the limited diagnostic sensitivity, this status reflects that the level of infection within the herd is below the detection limit but is not necessarily zero. By retesting every two years, these herds can maintain this status. In 2021, fifty-eight farms (35%) with 18,013 cows representing 14% of the cattle population in Thuringia were certified as ‘non-suspect’ [20,40]. In the affected herds, the programme focuses on reducing prevalence through hygiene measures to break the infection cycle and on reducing infection pressure through annual testing to detect and remove Map shedders. Hygienic measures are based on an initial veterinary risk assessment by specialised veterinarians and aim at establishing adequate hygiene improvement in a farm-specific manner. Recommendations are given on the basis of scientific evidence and considering the farmer’s needs and opportunities [20]. An evaluation of the programme in Thuringia concluded that control of Map was achievable at herd level provided that veterinary advice, sufficient testing capacities and financial aid were available to the farmers, and that re-introduction of Map was prevented [40].

In the Austrian province of Tyrol, a voluntary Map control programme was initiated in 2013 [41]. The programme is based on a two-step approach. The herd status is determined by biennial culture of boot swab samples; after two negative examinations, the herd is assigned an ‘unsuspected’ status. In test-positive herds, individual faecal samples of all cattle of 24 months of age and older are tested annually. Test-positive cattle have to be culled within 3 to 9 months after the positive test result, depending on the apparent prevalence in the herd [41]. In addition, preventive management measures are advised. If no positive individual results are obtained in a herd within a three-year period, the herd is assigned an ‘unsuspected’ status. Over half of the Tyrolean dairy herds, representing about 70% of dairy cattle, participate in the programme [21,42,43]. Results of the programme indicate a decreasing apparent herd prevalence: at the first (2013), second (2016/2017) and third (2018/2019) run of testing boot swab samples, 8%, 2% and 0.5% of herds tested positive, respectively [21,42,43]. The rapid decrease in the apparent prevalence may be related to the relatively small herd size of Tyrolean dairy herds in comparison to dairy herds in other countries [43].

### Recent and Anticipated Changes in Voluntary Programmes

In various countries, changes in the programme or the uptake of the voluntary programme were observed recently or are anticipated. 

In various countries, such as the Netherlands, Belgium, Denmark, Ireland, Italy, and the UK, ELISA testing of individual milk samples is being used to identify infected cattle in known infected herds [1,25,26,27,30]. In Ireland, the Irish Cattle Breeding Federation developed an interactive website on which participants have access to various overviews of test results on the herd level as well as on the individual animal level [44]. To support decision making by participating farmers with quarterly testing herds, a ‘Johne’s Progress Tracker’ was developed in the UK [38]. The Tracker monitors ten parameters related to the control of Map as indicators of the rate of new infections, the persistence of chronic infections, culling of test-positives and retention of infected cattle (Table 1) [25,38]. Benchmarks have been developed for each of the monitored parameters using results of 253 participating dairy herds [25]. For participating herds, the monitoring of the parameters is performed by the milk-recording organisations using centrally developed algorithms [25]. A new development is to identify ELISA-positive cattle as a ‘priority cull’ if they have two ELISA results with an S/P ≥0.6 and/or a single ELISA result with an S/P ≥ 1.0 [25]. This aligns reasonably well with the results of a survival analysis of 90,835 heifers after their first milk ELISA test in the Dutch MQAP [45]. In this study, ELISA results with S/P ≥ 1.0 were reported as positive, and all results with S/P < 1.0 were reported as negative, whereas the actual S/P ratio was not reported. In heifers with an S/P of their first milk ELISA classified as S/P ≤ 0.20, 0.20 < S/P ≤ 0.40, 0.40 < S/P ≤ 0.60, 0.60 < S/P ≤ 0.80 or 0.80 < S/P < 1.00, the probabilities of becoming high positive (S/P ≥ 1.00) within two years after the first milk ELISA were 2%, 8%, 16%, 18% and 51%, respectively. These results suggest that heifers with a first milk-ELISA S/P < 0.80 have a rather low short-term risk of progression of the infection [45].

In Australia, a shift from government regulation to assurance programmes organised by the industry has been taking place. The previous large programmes set up to control Map in sheep and cattle in the 1990s and 2000s have been discontinued. However, market assurance programmes for cattle, sheep and goats are still coordinated by the National Johne’s Disease Project. Western Australia was considered free of the cattle strain of Map (but not the sheep strain) until 2021 when it was detected in a beef herd. This detection led to changes in management within the state, as well as relaxation of the requirements for cattle entering the state. To facilitate low-risk trade of cattle between herds, herds across Australia are assigned a ‘Johne’s disease dairy score’ or a ‘Johne’s beef assurance score’, ranging from 0 (high risk) to 8 (low risk) [28].

In Canada, four regional paratuberculosis control programmes exist, each consisting of the following four components: education, farm-specific risk assessment and management plans, testing and research [15]. In three of the four programmes, participating herds are assigned a herd status. In the past, up to 60% of Canadian dairy herds participated in the programmes. However, a major drop in participation rates was observed after the first subsidised phase of the programmes ended in 2014. This drop was associated with a shift in producer focus from paratuberculosis control to other priorities, such as lameness and animal welfare issues [15].

In various other countries, the development and optimisation of control programmes was anticipated. In Switzerland, initiation of a voluntary control programme was anticipated in 2023 [46]. The voluntary control programme will be run in addition to the existing legal requirement to notify the government of clinical paratuberculosis cases detected (since 1995) and mandatory control measures imposed on herds with clinical cases (since 2015). The latter mandatory control measures include the disposal of milk and carcasses of clinical cases, a movement ban of all animals from the herd and an obligation to slaughter offspring born to infected animals before 12 months of age [47]. The voluntary programme aims to ensure national and international market access for Swiss milk and milk products. The initial assessment and surveillance of test-negative herds consist of biennial testing of bulk milk samples or pooled milk samples by ELISA for antibodies against Map. In test-positive herds, individual milk samples are tested annually. ELISA-positive cattle have to be culled within 18 months [46].

In Italy, several amendments to the guidelines to control Map were recently proposed [27]. These included expansion of the target species towards cattle, buffalo, goat and sheep, restriction of herd testing to serum ELISA rather than individual milk ELISA testing, recommendations not to test serum samples within three months of intradermal tests for *Mycobacterium bovis* infection and regular herd visits to detect clinical paratuberculosis cases [27].

An internal review of the Dutch MQAP was performed to identify potential areas of improvement to increase its efficacy [30,48]. In this review, progress made in controlling Map was summarised whilst the design of the MQAP was compared to programmes in other European countries. In all, 25 potential areas of improvement were identified. For example, it was suggested to include ‘low-risk trade of cattle’ within the objectives of the programme and to identify a subset of herds with a high probability of freedom of infection as a source for low-risk trade of cattle. At the same time, the herd status of a herd in which cattle are introduced from a herd of origin with a lower herd status should be reduced accordingly [30,48]. The list of 25 topics may serve as a checklist of items that might be considered in the design of paratuberculosis control programmes in other countries.

A recent evaluation of the programme in the German region Thuringia [40] concluded, in agreement with the Dutch review [48], that an easy-to-use risk-scoring system reflecting the potential herd-level risk of Map for potential buyers of cattle needed to be implemented. Such a scoring system might be implemented alongside improved and cost-efficient surveillance of herds with the preferred status ‘non-suspect’ [40]. 

## 6. Financial Aid and Compensation

Although compulsory control programmes and legal requirements to control paratuberculosis are absent in most countries, financial support or compensation to farmers is provided in several countries. In several countries, including Ireland and Germany, costs are shared between state departments, milk processors funds and/or animal disease funds [20,26]. Most programmes provide funded support for one or more operational aspects of the control programme. The components covered include costs of testing, the conduct of risk assessments, or compensation for culled animals. For example, sampling and testing were free of charge for cattle owners in Norway, Austria [12,42] and Switzerland [46] and partly subsidised in Belgium [32], Germany, and Ireland [20,26], while there was no subsidy for testing in the Netherlands [30]. Several activities like screening or education were funded by dairy processors in the UK and by provincial milk marketing boards in Canada [25].

A veterinary risk assessment is supported in Ireland and some regions of Germany [20,26] but neither in Italy nor the Netherlands [27,30]. If culling of diseased or Map-positive animals is mandated, compensation for the value of culled livestock and the cost of its culling is covered by the state and/or an animal disease fund in some countries. For example, in Austria and Switzerland, compensation is paid for clinically diseased animals that must be culled [42]. These incentives are meant to stimulate farmers to take control measures [47].

Financial support may have begun or ceased due to programme review processes. For example, in Australia, support was paid to beef farmers until 2015, then ceased [1], and in Canada, subsidised first phases of the programme ended in most provinces in 2014, and costs are now paid by the participating producers. In both countries, active participation dropped significantly when the financial support ceased, and the focus of the producers subsequently changed [15].

## 7. Compulsory Control Programmes

Notwithstanding the advantage of broad inclusion of all cattle farmers, compulsory control programmes are rare in the context of paratuberculosis. The two Scandinavian countries Norway and Sweden are unique in the world with their nationwide programmes aiming at eradication of paratuberculosis. In most countries, only some regions run compulsory programmes or mandatory measures [1,12]. 

Lower Saxony, a federal state of Germany, has implemented a mandatory control programme in 2017. Since then, testing of pooled milk samples (*n* = 50) for the presence of Map-specific antibodies has been compulsory in dairy herds. The pool-milk ELISA has to be repeated biennially, and in the case of positive pool-test results, ELISA tests on individual serum samples of cows (≥24 months) have to be performed. If at least one cow is identified as ELISA-positive, a risk assessment and management plan has to be written out by a veterinarian. Therefore, paratuberculosis control efforts contribute to a general improvement in herd biosecurity. Subsequently, in herds with ELISA-positive cattle, individual testing has to be performed annually. As soon as an apparent within-herd prevalence < 2% is reached, the farmer can switch back to biennial testing by pool-milk ELISA [20]. During the first 4 years after the implementation of the programme (2018–2021), about 2.05 Mio. samples were tested from an average number of 10,041 herds. About 10% of all tested farms in Lower Saxony had a within-herd Map apparent ELISA prevalence above 5% [20]. Furthermore, a negative pre-movement ELISA result is a mandatory requirement for the introduction of cattle into Lower Saxonian dairy herds. 

In Austria, another compulsory concept has been established by a national regulation where the obligation to prohibit clinical cases of paratuberculosis from entering the food chain was implemented in 2006 [49]. Any suspicion of clinical paratuberculosis at slaughter results in a requirement to test samples of liver tissue, ileocaecal lymph nodes and the small intestine by PCR, and if PCR-positive, there is a requirement to dispose of the carcass [49]. A similar approach was implemented in Switzerland in 2015 [1]. Suspicion of disease in clinically ill farmed ruminants must be reported, and in case of Map detection in samples taken by the competent authority, the animal must be culled within 3 days. In addition, in Switzerland, the offspring of Map-positive animals born within the previous 12 months of detection has to be slaughtered by the age of 12 months at the latest. The mandatory measures in Switzerland do not aim at the eradication of Map but are meant to eliminate high shedders to reduce the infection pressure on the farm and the entry of Map into the food chain [46].

Similarly, components of control are compulsory in several countries, according to the local situation and goals. For example, vaccination of sheep is mandatory in affected areas of Iceland [1], and compulsory active surveillance was established in Japan [50]. In several countries, mandatory measures are combined with voluntary components. Such a two-step programme was established in Lower Saxony, combining compulsory measures like surveillance, restrictions on animal movements and counselling of farmers that own a positive herd with voluntary tagging and culling of test-positive dairy cattle [20,41]. 

Another example of a compulsory component is the veterinary risk assessment and management plan (V-RAMP), which is mandatory for participating cattle farmers in both Northern Ireland and the Republic of Ireland [44,51]. The aim of this V-RAMP is to identify and correct aspects of management that could contribute to the introduction and/or spread of infection. This includes farm-specific paratuberculosis control training. A detailed annual risk assessment is carried out by a trained veterinary surgeon in partnership with the herd owner. About 200 veterinary practitioners are trained online by the Animal Health & Welfare Northern Ireland organisation. All large animal practices in the country have at least one trained vet. This mandatory counselling is combined with advisory annual testing of all animals over 2 years of age using a blood or milk sample or environmental screening sampling [51].

A compulsory element of animal disease control with respect to paratuberculosis is its passive surveillance as mandated by the new European Animal Health Law. This includes the obligation of farmers to notify the competent authority of any suspicion of paratuberculosis [52,53]. The competent authority must verify the suspicion and report confirmed cases annually to the European Commission [53,54]. As discussed in the ParaTB Fora, there is little experience regarding the efficacy and effectiveness of this notification system. 

## 8. Surveillance in Test-Negative (Meta)Populations

Surveillance of test-negative herds and flocks and test-negative metapopulations (such as all herds and flocks in a region or country) is important to avoid inadvertent spread of any introduced Map infections. In 2017, the International Association for Paratuberculosis (IAP) published a consensus statement with guidelines for certification with respect to the movement of livestock [55]. Given that a negative clinical history of Map infection has a low negative predictive value of herd or flock status, the guidelines stated that the likelihood that a country, region or herd/flock is not infected can only be demonstrated by ongoing negative herd or population testing and active surveillance on a large scale over long periods [55]. Potential area classifications included ‘free area’, ‘eradication area’, ‘certification area’ and ‘other area’.

In the IAP guidelines, a ’free area’ was defined as ‘a country, zone or compartment in which Map infection is notifiable and extensive and large-scale surveillance for Map infection by the animal health authority has not identified endemic infection for ten years, or where infection has been introduced it has been demonstrably stamped out by slaughter and intensive tracing of suspect infection and intensive surveillance has not identified Map for 2 years’. Furthermore, an ‘eradication area’ was defined as ‘A country, zone or compartment in which Map infection is at low prevalence, is notifiable and extensive and large-scale compulsory surveillance for Map infection by the animal health authority continues to demonstrate a low herd prevalence of infection and where the herd prevalence of infection is demonstrably being reduced as infection is stamped out’ [55]. As discussed above, paratuberculosis has not been detected in Norway since 2016 despite extensive surveillance [12]. Thus, Norway may at present qualify as an ‘eradication area’ according to the guidelines. Moreover, provided extensive surveillance does not detect any new infections in the coming years, Norway may qualify as a ‘free area’ from 2026 onwards.

According to the IAP guidelines, a ‘certification area’ was defined as ‘A country, zone or compartment in which an officially sanctioned and recognised voluntary herd or flock classification programmes, with a certification component based on sound farm biosecurity and negative herd and/or flock testing and surveillance operates to objectively classify herds and/or flocks for Map risk’ [55]. In Switzerland, a temporary movement ban is imposed on herds with laboratory-confirmed clinical paratuberculosis cases, but this is not followed up by active surveillance, including testing of cattle, other than an examination for clinical signs of paratuberculosis [46]. In fact, in none of the paratuberculosis control programmes presented at the 7th and 8th ParaTB Fora was extensive obligatory active surveillance combined with the full prohibition of the transfer of cattle from known test-positive herds to test-negative herds [15,20,24,25,27,28,30,42,46,56]. Thus, none of the areas in which these programmes are run may currently qualify as a ‘certification area’. 

At the herd and flock level, the IAP guidelines recommend that paratuberculosis programmes include a test strategy that appropriately documents a specific probability of freedom from infection [55]. Simulation studies indicated that whole-herd examinations by individual milk ELISA may be suitable to identify herds with a high probability of freedom from Map infection, i.e., a high probability of a within-herd prevalence below a design prevalence of 5%, even though eleven annual herd examinations are required to reach a 95% confidence of freedom in test-negative herds [57,58,59,60]. Similarly, test results of repeated environmental samples and pooled milk samples might be used to estimate the probability of freedom from Map infection in herds under surveillance [19,20]. In line with these results, a review of the Dutch MQAP recommended to include ‘low-risk trade of cattle’ within the objectives of the programme and to identify a subset of herds with a high probability of freedom from infection as a source of low-risk cattle for trade [48]. Predictive modelling to identify test-negative herds in the Dutch MQAP with a high risk of future positive test results was recently developed [61]. A similar approach might be used to identify test-negative herds with a low risk of future positive test results as a proxy parameter for disease freedom. In the German region Thuringia, the probability of freedom from infection was calculated from the results of various surveillance methods with different system sensitivities and the probability of Map introduction through introduction of cattle from other herds [62].

## 9. Discussion

This narrative review aimed to summarise recent information about paratuberculosis control programmes and their progress presented at the 7th and 8th IDF ParaTB Forum in 2021 and 2022 and at the 15th International Colloquium on Paratuberculosis in Dublin in 2022. In doing so, this review does not provide an extensive global overview but is restricted to the control efforts presented at these meetings. The IDF Fora are primarily focused on the dairy sector. Furthermore, programmes presented at the above meetings were run in developed countries, and reports from low- and middle-income countries are missing. However, the presented programmes varied from regional control activities to be implemented in the future to national control programmes that had been run for over 15 years. Therefore, key takeaways, identified gaps, and future perspectives from these programmes may be relevant to other control programmes in other countries as well.

### 9.1. Key Takeaways

A long-lasting stakeholder commitment is a prerequisite for a successful Map control programme because it requires a focus of the industry for 15–20 years to be able to show success and to measure the full impact. Therefore, short-duration projects of usually a 3-year time (and in some cases, optional extension for another 3 years) are not able to show the full potential benefit of sustained control efforts. From this perspective, the key takeaway was that programmes differ in progress and participation, depending on the stage of the life cycle of the programme, as well as on approaches in leadership, funding, and commitment across the value chain. On the one hand, in various European countries, Map control programmes are active with high participation rates. These programmes are characterised by incentives for farmers to participate and/or stable long-term funding implemented by milk processors or government bodies. Typically, these European programmes are managed by animal health organisations. The involvement of herd veterinarians in herd-level activities like testing, risk assessment and implementation of on-farm preventive management measures was reported to be an essential element to keep programmes going. Besides these core elements, subsidised cow-level ELISA tests and mandatory surveillance components were included in some of these programmes with a high uptake. On the other hand, outside Europe, Map prevention and control programmes were successfully started with reasonable participation but stopped after their first funding cycle because of the discontinued funding and a perceived lack of tangible benefit for farmers. Given the epidemiology of Map, the full potential of a Map control programme cannot yet be observed within the first 3–6 years of a funding cycle. Thus, North American milk processors did not buy into the programmes, leaving participating farmers and veterinarians without incentives to participate. In Canada, up to 60% of herds participated in one of the voluntary industry-supported regional control programmes, with numbers decreasing after funding ceased in 2014.

A second core message shown by many programmes was the realisation that the prevalence of Map can be reduced. The programmes in the Netherlands, the German regions of Lower Saxony, Saxony, and Thuringia, as well as in Tyrol and Northern Italy, observed a gradual decrease in the apparent animal-level prevalence of test-positive animals and in the proportion of high-risk herds over time. In the Netherlands, the proportion of herds with the preferred herd status increased from 45% to 80% within the first thirteen years of the initial assessment, while 91% of the initially test-positive herds progressed to the preferred herd status within this time frame [30]. In addition, results reported by Tyrol confirmed the outcome of a modelling study, which suggested that fadeout can be expected in a substantial percentage of infected small herds [43,63].

A strategy to increase the acceptance of Map control was the integration of Map control into generic programmes for improvement of biosecurity in cattle farms, which was reported to be successful for Australia, Canada, the UK and Germany [20,28]. This strategy may be applicable to other Map control programmes as well. In Australia, the quality assurance programme for cattle, sheep and goats has had a biosecurity module with an integrated optional Map section for cattle since 2017 [28].

Nearly all successful Map control programmes were accompanied by concurrent and practical research. Most of the programmes were initially implemented as pilot studies and guided by research teams. Research on several topics like testing, risk factors or participation has been shown to be vital not only during the implementation phase of the programme but also for evaluation and further development. Therefore, cooperation between universities, governmental institutes, producer organisations and other stakeholders is pivotal. Results, strengths, and challenges should be communicated to all stakeholders to keep motivation up. Not including a follow-up in a control programme is a missed chance because the presentation of programme data keeps farmers interested and provides an excellent opportunity to motivate them to keep Map on the radar screen and to gain a feeling for what is going on [20,24,30,42]. An example of evaluation and improvement is the Map control programme in Thuringia, which was implemented in 2003 and amended in 2008, 2014 and 2022 [20,40,64]. 

Interestingly, an important issue in starting successful Map control is the appropriate timing of the initiation of the programme to gain the right momentum. Agreement between all stakeholders to ‘get the wheel going’ is a prerequisite. Map control programme managers from several countries experienced only a limited window of opportunity to implement a programme and commented, ‘If you do not get it right, the window closes again, funding dies’ [15,65].. To predict or prepare the right context for starting is difficult because of unforeseeable changes in the prioritisation of resources, legal requirements, the perception of success and failure, as well as the occurrence of unexpected circumstances. Therefore, when the window of opportunity is open, a move forward should be made vigorously.

### 9.2. Gaps

In several programmes, important gaps were identified. Firstly, communication among all stakeholders was identified as pivotal for a successful control programme. The impact of Map infection must be quantified and broadcast. In most programmes, the potential association of Map with Crohn’s disease was identified as a main driver for Map control. However, for farmers and other stakeholders, this criterion is often insufficient to adopt and stimulate long-term on-farm Map control measures. Therefore, the benefits of control directly concerning farmers should be quantified to increase participation, e.g., reducing economic losses, increasing herd health, and improving animal welfare [3]. Further research into the motivation and barriers to participate may assist in increasing the uptake of programmes amongst farmers [66]. In addition, effective communication between all stakeholders is essential to achieve a common understanding of the progress made in the control programme, epidemiology and pathogenesis of the infection and disease, the basic functionality of useful management measures and the interpretation of test results. Tools for farmers and vets to track and integrate diagnostic information and risk assessment outcomes with an easy-to-capture visualisation of these results are observed to be helpful. The Johne’s Tracker of the UK National Johne’s Management Plan and the website of the Irish Johne’s control programme are good examples of how to present available information in a useful way [25,26].

Secondly, in most Map control programmes, little attention has been paid so far to transmission routes like intrauterine transmission, calf-to-calf transmission and adult-to-adult transmission, potentially contributing to a lack of long-term success in programmes that focus on dam-calf transmission and the removal of test-positive animals alone [8]. Although risks of introduction of cattle from other herds are commonly considered in risk assessment and management plans, these risks may be further addressed by compulsory post-movement testing and lowering the herd status to the status of the herd of origin if the herd of origin does not have the preferred herd status [48]. Moreover, given that multiple strains of Map can be present in infected herds [67], reducing the risk of the introduction of cattle is important in presumed uninfected herds and in known infected herds alike.

Thirdly, a genetic basis of susceptibility to Map infection has been identified [68,69,70]. Heritability estimates of susceptibility to MAP infection range from <0.01 to 0.30 [68,71,72,73,74,75,76,77,78], suggesting that breeding for disease resistance against MAP may be feasible. This is supported by the detection of genes and genetic loci associated with susceptibility to MAP infection [79,80,81,82,83,84,85,86]. A major problem in investigating the influence of genetics on susceptibility to MAP infection is the difficulty in accurately classifying susceptible, resistant or tolerant animals [8]. The ultimate goal of breeding for disease resistance is a reduction in the transmission of MAP. Thus, selection of breeding stock is attractive only if it results in offspring with reduced shedding of MAP, given exposure to MAP. Genome-wide association studies, which use various phenotypes to determine infection status (serology, faecal culture, tissue culture, pathology), have identified different chromosomes and chromosomal regions involved in resistance to MAP [84,87,88]. Studies using ELISA-based phenotyping can identify genes involved in the immune response to the agent, while phenotypes based on faecal or tissue culture identify genetic loci involved in the persistence of MAP infection, the development of granulomatous enteritis, and the shedding of MAP in faeces [87]. However, a high genetic correlation of 0.81 was observed between milk ELISA results and faecal shedding, indicating that selecting bulls based on their estimated breeding value for milk ELISA results is likely to result in offspring that are less likely to become infectious [89].

Three methods for utilising genetic variation in paratuberculosis control exist. The first involves deriving estimated breeding values from test results obtained in control programmes and using these breeding values to select pedigree cattle. However, the confidentiality of laboratory results of samples submitted by individual farmers presents a challenge that must be addressed to enable breeding organisations to make use of available test results. The second approach involves using genetic markers linked to resistance against (the progression of) Map infection to select breeding stock [85]. However, this approach faces a challenge as different genes are associated with different stages in the infection and disease process [8]. Therefore, a combination of various genetic markers is necessary. Before genetic markers can be used to guide the selection of breeding stock, it is necessary to rule out any potential undesirable genetic link with production traits [8]. A third route is to identify biomarkers for detecting resilient cattle that do not become infectious given Map infection and may eventually recover from infection [90,91,92]. However, to our knowledge, none of these three routes has been used in a paratuberculosis control programme. Therefore, the potential of genetic selection as a means of controlling Map should be further investigated and subsequently implemented in the near future.

Finally, at present, an official disease status regarding paratuberculosis for herds at a European or global level is missing. The World Organization for Animal Health (WOAH) has the mandate from the World Trade Organization (WTO) to officially recognise disease-free areas of countries for trade purposes. Currently, terrestrial animal disease-free areas were proclaimed for six diseases at the WOAH level and for eight diseases at the EU level [93]. Unfortunately, paratuberculosis is missing. This lack of regulation jeopardises the successes of regional Map control programmes because movement of infected animals or contaminated animal feed may pose a risk for (re-)introduction of Map [1]. A template for guidelines to establish international trade certifications for Map was provided by the International Association on Paratuberculosis in 2017 [55]. 

### 9.3. Future Perspectives

Considering the key takeaways and the identified gaps in paratuberculosis control, potential areas of improvement of existing programmes were identified, and new ideas may be adopted in both existing and newly developed Map control programmes [48]. However, given the diversity of the farming industry, variation in Map prevalence and variation across countries in the stage of the life cycle, maturity, and duration of Map control programmes, a ’one size fits all’ approach is likely to be ineffective.

A high uptake of Map control programmes amongst farmers is pivotal to achieving a beneficial effect on Map control in the metapopulation, such as a region or country. In various European countries like Belgium, the Netherlands and the UK, incentives to participate instigated by dairy processors were effective in achieving such a high uptake [25,32,33,35,38]. Furthermore, a strong and continued collaboration between official veterinary services, laboratories, veterinary practitioners, and farmers, as reported from Italy and Germany, is helpful [20,27]. Particularly in a voluntary setting, recruiting of participants and keeping them on board is pivotal. Mandatory elements like testing in Lower Saxony may be helpful, but their implementation needs political support. The inclusion of Map control into a broader biosecurity approach like in Australia, Canada, Germany, or in the style of the UK Red Tractor initiative (a general agricultural quality assurance programme) may help to reach a broader acceptance among farmers and stakeholders [20,25].

Keeping participants active is important. In addition to test-and-cull at regular intervals, performing periodical veterinary risk assessments like in the Irish programme [26] or regular meetings may help to keep farmers and stakeholders motivated and focused. Farmers’ and stakeholders’ motivation is strongly supported by perceived benefits from participation in the programme, such as improved animal health, certification of herds leading to financial trade benefits, and increased returns from the low-risk trade of cattle sold from herds with a preferential herd status.

Ease of sampling lowers the threshold for farmers to embark on Map control. Easy-to-use surveillance systems performed by a regular individual milk ELISA are already in place in Denmark, the UK, the Netherlands and Lower Saxony [20,25,30,94]. In addition, in previously test-negative herds, a confirmatory faecal assay of ELISA-positive cattle may be advised to confirm Map shedding and, thus, Map infection in the herd [27]. As an alternative testing approach, environmental samples can be tested for Map as is applied already in Tyrol and some regions of Germany [42,43] and was suggested in the framework of a two-step testing approach [20,41].

Estimation of the probability of freedom from Map infection [55] is a helpful tool for test-negative herds in low-prevalence populations. The probability of freedom can be derived from the probability of a herd becoming infected through introduction of cattle and the sensitivity of surveillance components such as repeated herd tests [13,20,58,60]. In low-prevalence populations, risk-based surveillance that involves testing of herds that are connected to Map-positive herds may be advantageous [56]. In contrast, in high-prevalence populations, an estimate of the true within-herd prevalence of Map infection might be more relevant than the probability of freedom from Map infection. However, to be able to compare the results of the different testing schemes, the herd-level sensitivity of the combined applied surveillance components has to be determined [13,95,96].

Finally, since animal trade is common, mitigating the risk of spreading Map between herds is pivotal for successful control in every stage of a control programme. Thus, the use of animal movement data to inform the risk profile of herds participating in a Map control programme is a crucial element in improving Map control. In most developed countries, this information is available from central databases and should be made officially accessible for Map control to reduce the risk of introducing Map-positive cattle into low-risk herds. 

## 10. Conclusions

Paratuberculosis control programmes have been implemented in several countries with a relevant cattle industry. These programmes vary largely in maturity and duration, uptake amongst livestock farmers and progress made in Map control. 

In some countries, pre-existing Map control programmes were integrated into generic biosecurity programmes. Notwithstanding variation among programmes, various Map control programmes were observed to result in a gradual decrease over time in Map prevalence and the proportion of high-risk herds. The successful control of Map in Norway illustrates that, under the right conditions, elimination of paratuberculosis at the herd or flock level and eradication from a metapopulation are achievable. 

Various future perspectives on Map control were identified. Improved communication targeting drivers and barriers to participation may stimulate the entry and retainment of farmers in control programmes as well as support the implementation of control measures in their herds. Additional control measures focusing on vertical transmission, calf-to-calf transmission and adult-to-adult transmission may improve the efficacy of control in infected herds. 

Implementation of compulsory post-movement testing and of disincentives to introduce cattle from high-risk herds may stimulate a risk-averse behaviour of farmers when purchasing cattle. The risk profile of herds of origin can be derived from the probability of freedom from Map infection and an estimated true within-herd prevalence of Map. A valid surveillance scheme is required to inform such risk profiles. Easy-to-use surveillance systems by a regularly performed individual milk ELISA have been incorporated into various Map control programmes. Recently, easy-to-capture visualisation of test results to inform farmers on the progress of Map control in their herds was included in some programmes. On the metapopulation level, results of the surveillance schemes may be used to inform genetic selection for resistance to Map infection as well. 

In conclusion, at the recent Paratuberculosis Fora and International Colloquium on Paratuberculosis, it was demonstrated that a reduction in Map prevalence can be achieved by Map control programmes. Various prerequisites for successful programmes, gaps in current programmes as well as perspectives for future improvements of the programmes were identified.

## Figures and Tables

**Table 1:** Parameters related to the control of Map that are monitored in herds participating in the UK National Johne’s Management Plan [25,38].

Factor in the Control of Map	Monitored Parameters	25%, 50% and 75% Percentiles ^1^
Progression of Map (new infections)	Proportion of tested cattle testing positive for the first time	1.6%; 2.4%; 3.2%
	Proportion of tested cattle testing positive for the first time amongst all parity 1 cattle present	0.9%; 1.8%; 3.0%
Persistence of Map (chronic infections)	Proportion of tested cattle with ≥2 out of 4 consecutive tests being positive	2.1%; 3.8%; 5.9%
Removal (culling of test positives)	The relative risk of removal within 150 days of cattle with ≥2 out of 4 consecutive tests being positive in comparison to test-negative cattle	1.81; 2.69; 4.07
Retention (breeding)	The relative risk of serving cattle with ≥2 out of 4 consecutive tests being positive in comparison to test-negative cattle s	0.24; 0.55; 0.81
	The relative risk of calving cattle with ≥2 out of 4 consecutive tests being positive in comparison to test-negative tested cattle	0.52; 0.74; 0.97
Overall measures of the control of Map	Average S/P ratio of a whole milking herd screen	0.075; 0.092; 0.108
	Proportion of results with an S/P > 0.3	2.9%; 4.7%; 6.5%
	Proportion of results with an S/P > 0.6	0.7%; 1.4%; 2.7%
	Proportion of results with an S/P > 1.0	0.0%; 0.4%; 1.1%

^1^ Based on observations in 253 herds with at least seven whole herd milk ELISA tests during 2019 and 2020 using the IDEXX milk ELISA with a cut off S/P ratio of 0.30.

## Data Availability

No new data were created or analyzed in this study. Data sharing is not applicable to this article.
